# The Influence of Cigarette Smoking on Gingival Bleeding and Serum Concentrations of Haptoglobin and Alpha 1-Antitrypsin

**DOI:** 10.1155/2013/684154

**Published:** 2013-10-29

**Authors:** Fouad H. Al-Bayaty, NorAdinar Baharuddin, Mahmood A. Abdulla, Hapipah Mohd Ali, Magaji B. Arkilla, Mustafa F. ALBayaty

**Affiliations:** ^1^Center for the Study of Periodontology, Faculty of Dentistry, Universiti Teknologi MARA (UiTM), 40450 Shah Alam, Selangor Darul Ehsan, Malaysia; ^2^Faculty of Dentistry, University of Malaya, 50603 Kuala Lumpur, Malaysia; ^3^Department of Molecular Medicine, Faculty of Medicine, University of Malaya, 50603 Kuala Lumpur, Malaysia; ^4^Department of Chemistry, Faculty of Science, University of Malaya, 50603 Kuala Lumpur, Malaysia; ^5^Faculty of Medicine, University of Malaya, 50603 Kuala Lumpur, Malaysia

## Abstract

The objectives of this study were to evaluate the influence of cigarette smoking on gingival bleeding and serum concentrations of cotinine, haptoglobin, and alpha 1-antitrypsin in Malaysian smokers. A total of 197 male smokers and nonsmokers were recruited for this study. Plaque index, bleeding on probing (BOP), and levels of serum cotinine, haptoglobin, and alpha 1-antitrypsin were evaluated. The data were analyzed using SPSS version 20.0, with the significance level set at *α* ≤ 0.05. Linear regression analyses were performed. The mean cigarette consumption per day was 13.39 ± 5.75 cigarettes; the mean duration was 16.03 ± 8.78 years. Relatively low BOP values (26.05 ± 1.48) and moderate plaque indexes (51.35 ± 11.27) were found. The levels of serum cotinine (106.9 ± 30.71 ng/dL), haptoglobin (76.04 ± 52.48 mg/dL), and alpha 1-antitrypsin (141.90 ± 18.40 mg/dL) were significantly higher in smokers compared to non-smokers. Multiple logistic regression models for all variables and smokers demonstrated observed differences between BOP, the number of cigarettes per day, and duration of smoking, while serum cotinine, haptoglobin and alpha-1 antitrypsin levels showed no significant differences. Duration of smoking (years) and the cotinine level in serum showed a significant correlation with plaque index. The present analysis demonstrated that the duration of smoking in years, but not the number of cigarettes smoked per day, was associated with reduced gingival bleeding in smokers.

## 1. Introduction

Numerous cross-sectional studies demonstrate increased prevalence of periodontal disease among smokers [1–3]. Long-term prospective studies demonstrate that smoking worsens the periodontal condition in smokers compared to those who have quit smoking and to nonsmokers.

Cigarette addiction remains a harmful habit that facilitates the development and progression of periodontal diseases, even when other contributory factors, such as oral hygiene, plaque, calculus, and socioeconomic and demographic issues, are in check. The associated risk was estimated with odds ratios of 2.5 to 6.0 [2, 4, 5]. The connection that links smoking and periodontal diseases is based on the number of cigarettes. The probability for further attachment loss varies from 2.05 for light smokers to 4.75 for heavy smokers. This result conforms with the hypothetical idea that smoking has increasing consequences on periodontal health [6].

Gingival bleeding upon probing (BOP) provides a quantitative indication of gingival/periodontal inflammation. A longitudinal study into the association between BOP and loss of attachment showed that bleeding on probing presents diagnostic predictability in treated patients. BOP is an indicator for disease progression, but it is by no means definitive. Nevertheless, it is a vital indicator in the assessment of the degree, as well as the extent, of disease in a periodontal patient [7]. 

Clinically, smokers presented with reduced signs of inflammation compared to nonsmokers [8, 9]. This result can be due to the temporary gingival vasoconstriction induced by nicotine. The swollen gingiva in smokers that exhibits decreased vascular density and angiogenesis compared with that of nonsmokers suggests a suppressed inflammatory response that accounts for impaired wound healing [10]. Chronic smokers present with reduced gingival BOP, which masks the clinical markers often used by dentists to monitor periodontal health [8]. Traditionally, reduced bleeding in smokers has been attributed to gingival vasoconstriction induced by the actions of nicotine-stimulated adrenaline; however, the available evidence that supports this hypothesis in humans is not conclusive as smoking can cause vasodilatation in some tissues due to the action of noradrenaline on A1-adrenergic receptors. There is some evidence to support this theory in animal models [3]. Serum cotinine concentrations were chosen to test the hypothesis that nicotine (cotinine) decreases gingival bleeding because of its vasoconstrictive effects, which are mediated through norepinephrine. Many components of cigarette smoke are able to alter the function of immune cells [11, 12]. Acute phase reactants (APRs) are the markers of inflammation, which are synthesized in response to tissue damage and inflammation. During inflammation, their concentrations increase one thousandfold over the normal levels [11]. These markers are mainly produced by hepatocytes, as well as by adipocytes, fibroblasts, and endothelial cells. Similarly, the levels of cytokines and acute phase proteins (APP) in plasma are due to tissue injury and inflammatory conditions along with lower levels of all mediators in healthy nonsmokers (HNS) versus smokers (HS), signifying that these systemic inflammatory markers changed in response to a threat by harmful materials from smoking [12]. 

The concentration of cytokines and acute phase proteins (APP) in plasma notably alters due to tissue injury, infection, burn, shock, several types of inflammatory conditions, and cancer. Smoking shows a relationship with acute phase proteins similar to C-reactive protein (CRP) and fibrinogen [13]. Smokers show increased levels of circulating leukocyte counts and inflammatory markers, including CRP, intercellular adhesion molecule Type-1, interleukin (IL-6), E-selectin, and P-selectin [14]. Alpha 1-antitrypsin (A1AT) is formed in the liver, and it participates in protecting the lungs from neutrophil elastase, a particular enzyme that is capable of disrupting connective tissue. The normal blood levels for alpha 1-antitrypsin are within the range of 81–171 mg/dL. This range confers protection to tissues against enzymes of inflammatory cells, particularly neutrophil elastase, although the concentration can increase upon acute inflammation. In smokers, the levels of alpha 1-antitrypsin were exclusively and considerably raised and were related to the extent of smoking [15].

Haptoglobin is generated mainly by hepatocytes and some other tissues, including the skin, lung, and kidney. The normal range is 33–171 mg/dL. As it is certainly an acute-phase protein, any inflammatory condition linked to infection, extreme stress, burns, major crush injury, allergy, and so forth will possibly boost the levels of plasma haptoglobin. In smokers, the levels of haptoglobin increased with the degree of smoking [16]. Haptoglobin and alpha 1-antitrypsin were not previously evaluated in Malaysia's smoker population. We hypothesize that smoking is associated with decreased gingival bleeding in a dose-dependent manner. The objective of this study is to evaluate the influence of cigarette smoking on gingival bleeding and on the serum concentrations of haptoglobin and alpha 1-antitrypsin in selected Malaysian smokers.

## 2. Materials and Methods 

Convenience samples of 197 (99 smokers and 98 nonsmokers) male patients were recruited for this study. They were either patients or those accompanying them to the Primary Care Unit (PCU), Faculty of Dentistry, at the University of Malaya. All participants were free of any systemic diseases, and they were not on any medication for the last 6 months. Aspirin, nonsteroidal anti-inflammatory, and nicotine replacement medications were not used by the participants; bleeding tendencies, such as von Willebrand disease, were not assessed in the participants. The mean age was 40.4 ± 12.0 (20–64) years. The nature of the study was explained to all patients. They were required to sign a written consent form prior to the commencement of the study.

This study was in agreement with the ethical principles of the World Medical Association's Declaration of Helsinki (1964). The study was approved by the Research and Ethical Committee of the University of Malaya DF OP0701/0003(L). The respondents were requested to fill out a questionnaire with their health-related records as well as their history of cigarette smoking. 

The survey was completed independent of the medical testing. The criteria used for the assessment of smoking status were performed according to the criteria established by the US Center for Disease Control and Prevention (CDC). Current smokers were defined as those who had smoked over 100 cigarettes over their lifetime and who were smokers at the time of the interview. Former smokers were those who smoked over 100 cigarettes in their lifetime but were not currently smokers, while nonsmokers were those who had not smoked over 100 cigarettes in their lifetime [17].

All participants were examined by one calibrated expert examiner. Full mouth (except for molars) recording for visible plaque index and bleeding on probing (BOP) introduced by Ainamo and Bay was performed [18]. For the visible plaque index, the patient was asked to rinse with plaque-disclosing solution (Chrom-O-Red disclosing solution, Germiphene, Brantford, ON, Canada) prior to recording. For BOP, all teeth were air dried with the air syringe. 

Intraoral examination was performed and WHO dental probe was used to assess bleeding on probing and dental plaque, and BOP was documented as absence or presence (0/1). Bleeding was counted as a dichotomy of variables at every location (present/absent). The manifestation of bleeding in 10 seconds indicated a positive score that was expressed as a percentage of the total number of gingival margins examined. Intraexaminer calibrations performed on plaque index (PLI) and bleeding on probing (BOP), 180 sites, were measured on five patients, and the records were charted. One and a half hour following the initial measurements, the examiner remeasured the same 180 sites, and the records were charted. The charting was done again two times on the same patients. Data were analyzed with SPSS and Cohen's Kappa coefficient. The result of the analysis was Kappa = 0.81 (*P* < 0.001), which showed almost perfect agreement [19].

A 10 mL blood sample was obtained in a vacutainer tube containing EDTA for analysis. All blood samples were centrifuged, and the serum was aspirated in new tubes and preserved in a refrigerator under −20°C until being subjected to laboratory analysis. The serum cotinine was analyzed by the ELISA technique (ELISA Kit, Sigma Aldrich, USA). The limit of detection and the upper limit for linearity for the serum cotinine assay were 1 ng/mL and 500 ng/mL.

 Haptoglobin and alpha 1-antitrypsin protein levels were measured using the Beckman Coulter Image Immunochemistry System (Sigma Aldrich, USA). Clinical examination and clinical parameters were recorded over several months; additionally, the entire laboratory test was conducted over two weeks.

## 3. Statistical Analysis

All analyses were performed with SPSS for Windows version 20.0 (Chicago Inc.). The significance level was set at *α* ≤ 0.05 with a 95% confidence interval. The obtained data were checked for completion, and a double entry method was used to ensure error-free data. Preliminary organization of the data was performed, and the results were presented in three steps. One step was the descriptive statistics of the study subjects; the second step was the univariate correlation and multiple linear regression model. The data were summarized using means, corresponding standard deviations and 95% confidence intervals. The independent *t*-test was used to compare smokers and nonsmokers. A scatter plot correlation analysis was employed to test the association between the outcome variables and the predictor variables. Variables that are significant at 5% were identified and included in the final regression model. Linear regression analysis was performed for BOP and the PI, and the respective variables were significant after bivariate analysis.

## 4. Results

A total of 197 male smoker and nonsmoker patients participated in this study. There were 23640 sites examined to assess the visible plaque index and bleeding on probing.  Table 1 gives an overview of the means, standard deviations, and 95% confidence intervals (CI) of the variables evaluated in this study. The mean cigarette consumption per day was 13.39 ± 5.75 sticks; the mean duration was 16.03 ± 8.78 years. Smokers demonstrated a relatively low mean bleeding index of 26.05 ± 1.48 and a higher plaque index of 51.35 ± 11.27 compares to nonsmokers, 26.29 ± 17.219 and 51.14 ± 18.64. Statistical analysis revealed nonsignificant deference. *P* values were 0.941 and 0.939, respectively. 

The serum cotinine level in smokers was 106.9 ± 30.71, relatively the same level as that found in smoker by previous researches and higher than that found in nonsmokers, 19.20 ± 8.59. Statistical analysis demonstrated significant deference. *P* value was 0.000.

Haptoglobin and alpha 1-antitrypsin (76.04 ± 52.48 and 141.90 ± 18.40 mg/dL) showed higher means than those evaluated in nonsmokers. Statistical analysis demonstrated high significant deference. *P* value was 0.000.

Table 2 represents the association between BOP and other variables. In a preliminary main effect multiple logistic regression model for all variables and smoker subjects, the only significant differences were found between BOP and the number of cigarettes per day and between BOP and the duration of smoking (years) where *P* value was 0.001, while the serum cotinine, haptoglobin, and alpha 1-antitrypsin levels showed no significant differences where *P* values were 0.039, 0.144, 0.198, respectively. However, smoking was negatively associated with gingival bleeding; only the duration of smoking was significantly related to BOP at regression analysis as *P* value was 0.004 (Table 3).

Table 4 shows a correlation between plaque index and smoking variables. A nonsignificant correlation was found between the number of cigarettes per day and the haptoglobin and alpha 1-antitrypsin levels where *P* values were 0.870, 0.178, and 0.052, respectively, while the duration of smoking (years) and the cotinine level in serum showed a significant correlation to plaque index where *P* values were 0.001 and 0.001, respectively. Regression analysis for plaque index and factors revealed significant differences for the duration of smoking (years) and the cotinine level *P* values were 0.001 for both (Table 5).

## 5. Discussion

Cotinine is the preferred serum biomarker for tobacco exposure; its levels can be used as an objective, reliable and quantitative method to evaluate the role of smoking in inflammatory periodontal disease [20]. 

Both local and systemic effects of cigarette smoking on periodontal diseases have been suggested [21, 22]. Gingival bleeding and vascular hyperemic reactions linked with plaque-induced gingivitis are reduced in smokers. The difference in bleeding responses between smokers and nonsmokers has generally been attributed to the vasoconstrictive properties of nicotine [23, 24].

The main purpose of the current study is to examine the relationship between cigarette smokers and overt signs of gingival inflammation, such as bleeding upon probing, in, otherwise, healthy smokers. For this reason, patient selection was based largely on the extent of the smoking status and it did not consider the periodontal status of the patients as a number of earlier studies [10, 25] selected patients who were diagnosed with gingival inflammation. This assortment was mostly chosen to evaluate the relationship between smoking and gingival bleeding and to enhance understanding about the connection of smoking to gingival bleeding. The period of smoking in these cases ranged from 5 to 33 years, which was afterwards computed into pack years. However, some earlier studies [3, 26] classified people who had quit smoking for approximately 2 to 5 years or more under the nonsmokers group. It was decided in this study to exclude former smokers to eliminate any long-term effects of smoking on periodontal tissues. Females were purposely excluded from the study because it is difficult to recruit females who admit that they smoke. The second rationale for not including females was to avoid potential hormone-induced microcirculatory changes [27]. 

Smokers demonstrated a relatively low mean bleeding index, a higher plaque index, and high cotinine, alpha 1-antitrypsin, and haptoglobin levels compared to nonsmokers, possibly due to the local and systemic effects of smoking on these parameters [8, 9, 14].

The oral hygiene status, as represented by plaque scores, for smokers was 51.35 ± 11.27, and regression analyses demonstrated a significant relationship between plaque score, duration of smoking (years), and serum cotinine. This finding is in accordance with earlier studies [3, 28, 29]. However, other studies showed higher plaque levels in smokers [30, 31], while some studies demonstrated less plaque levels in smokers [32, 33]. In the present analysis, the results showed that gingival bleeding was not affected by the number of cigarettes smoked per day. The findings of our study do not appear to support the fact that the number of cigarettes per day suppresses gingival bleeding (Table 3). These findings were in disagreement with the results of several studies [9, 34]. Only the duration of smoking in years suppressed the effect on gingival bleeding upon gentle probing (−0.200 and 0.997).

The relationship between smoking and gingival bleeding appears to be dose dependent. The mean number of cigarettes smoked daily was 13.39. This result is in concurrence with earlier studies that investigated the dose-response function among current smokers [24]. The present data suggest that lifetime exposure to smoking exerts an effect on reducing bleeding on probing. This work is in agreement with previous studies [7, 35] that demonstrated reduced gingival bleeding in relation to duration in years. The mechanisms by which smoking suppresses gingival bleeding are not well understood. Usually, decreased bleeding in smokers is linked to gingival vasoconstriction aggravated by the effects of nicotine-stimulated adrenaline, as well as noradrenaline, on *α*1-adrenergic receptors.

Smoking has profound consequences on the immune and inflammatory systems [36]. Smoking has adverse effects on fibroblast function [37], chemotaxis, and phagocytosis by neutrophils [38] and immunoglobulin production [39].

Tissue injury occurs mainly due to oxidative stress and also indirectly through the activation of redox-sensitive gene transcription factors, such as nuclear factor-kappa beta [30], which subsequently navigates downstream proinflammatory cytokine/chemokine production [40]. The resultant periodontal inflammation creates a low-grade inflammatory response that is detectable within the peripheral vasculature [41]. 

The evaluation of serum acute phase proteins may help in identifying patients who are at high risk for destructive disease or reveal those patients who are undergoing a process of periodontal breakdown. An increasing number of studies are focusing on the relationship between C-reactive protein and periodontitis. A high plasma level of this protein was observed in patients with severe periodontitis in comparison to the healthy group [42]. Our results showed no significant relationship of haptoglobin and alpha 1-antitrypsin with smoking duration, bleeding on probing, plaque index, and cigarette consumption per day. On the basis of the observation that smokers may present with a lower level of gingival inflammation, it has been speculated that the gingival blood flow in smokers may be less in comparison to nonsmokers [8, 10]. This would also induce a decreased local host response. So, smoking is thought mainly to affect the periodontal tissues by way of the vascular and immunological response of the body. The demonstration of correlations of levels of immunosuppressive acute phase proteins with smoking extent and age among normal subjects suggests that changes in the levels of these proteins may be related etiologically to the association of smoking [15]. Further study is required to evaluate the significance of bleeding on probing before and after nonsurgical treatment in smokers.

## 6. Conclusions

In conclusion, the present analysis demonstrated that the duration of smoking in years was associated with reduced gingival bleeding and plaque formation in smokers; haptoglobin and alpha 1-antitrypsin levels were higher in smokers. The cotinine level in serum showed a significant correlation with plaque index.

## Figures and Tables

**Table 1:** The mean standard, deviation, and 95% confidence intervals (CI) of the variables evaluated in the study in both smokers and non-smokers.

	Smokers			Nonsmokers			*P* value	S/NS
	Patients number	Mean ± SD	95% CI	Patients number	Mean ± SD	95% CI		
Number of cigarettes per day	99	13.39 ± 5.75	12.25, 14.54	—	—	—	—	—
Duration of smoking per years	99	16.03 ± 8.78	12.92, 19.14	—	—	—	—	—
BOP	99	26.05 ± 1.48	23.09, 28.99	98	26.29 ± 17.219	−8.16, 7.58	0.941	NS
Plaque index	99	51.35 ± 11.27	47.35, 55.35	98	51.14 ± 18.64	−7.14, 7.72	0.939	NS
Cotinine (ng/dL)	99	106.9 ± 30.71	96.04, 117.82	98	19.20 ± 8.59	76.56, 98.97	0.000	S
Haptoglobin (mg/dL)	99	76.04 ± 52.48	57.24, 94.84	98	32.46 ± 6.30	24.66, 62.48	0.000	S
Alpha 1-antitrypsin (mg/dL)	99	141.90 ± 18.40	135.38, 148.43	98	123.57 ± 12.20	10.71, 25.97	0 .000	S

**Table 2:** Association between BOP and other variables.

	Mean ± SD	Patient number	Pearson correlation	*P* value	S/NS
Number of cigarettes per day	13.39 ± 5.75	99	0.323	0.001	S
Duration of smoking in years	16.03 ± 8.78	99	0.432	0.001	S
Cotinine (ng/dL)	106.9 ± 30.71	99	0.039	0.708	NS
Haptoglobin (mg/dL)	76.04 ± 52.48	99	0.144	0.155	NS
Alpha 1-antitrypsin (mg/dL)	141.90 ± 18.40	99	0.198	0.049	NS

**Table 3:** Regression analysis of BOP and factors significant at the univariate level; only duration of smoking is significantly related to BOP at regression analysis.

Model	Beta	*P* value	95% CI	S/NS
Constant	—	0.552	−15.147, 28.175	
Number of cigarettes per day	0.104	0.361	−0.312, 0.848	NS
Duration of smoking in years	0.352	0.004	−0.200, 0.99799	S
Alpha 1-antitrypsin mg/dL	0.055	0.575	−0.113, 0.202	NS

**Table 4:** Correlation between plaque index and smoking variables.

	Mean ± SD	Patient number	Pearson correlation	*P* value	S/NS
Number of cigarettes per day	13.39 ± 5.75	99	0.017	0.870	NS
Duration of smoking in years	16.03 ± 8.78	99	0.366	0.001	S
Cotinine (ng/dL)	106.9 ± 30.71	99	0.263	0.008	S
Haptoglobin (mg/dL)	76.04 ± 52.48	99	0.137	0.178	NS
Alpha 1-antitrypsin (mg/dL)	141.90 ± 18.40	99	0.196	0.052	NS

**Table 5:** Regression analysis for plaque index and factors.

Model	Beta	*P* value	95% CI	S/NS
Constant	—	0.001	48.703, 63.633	
Duration of smoking in years	0.438	0.001	0.333, 0.792	S
Cotinine (ng/dL)	−0.353	0.001	−0.195, −0.064	S

All of the variables are significant and protective.
